# Green Synthesis of Silver Nanoparticles by the Cyanobacteria *Synechocystis* sp.: Characterization, Antimicrobial and Diabetic Wound-Healing Actions

**DOI:** 10.3390/md20010056

**Published:** 2022-01-06

**Authors:** Nancy S. Younis, Maged E. Mohamed, Nermin A. El Semary

**Affiliations:** 1 Pharmaceutical Sciences Department, College of Clinical Pharmacy, King Faisal University, Al-Ahsa 31982, Saudi Arabia; Memohamed@kfu.edu.sa; 2 Pharmacognosy Department, College of Pharmacy, Zagazig University, Zagazig 44519, Egypt; 3 Biological Sciences Department, College of Science, King Faisal University, Al-Ahsa 31982, Saudi Arabia; nelsemary@kfu.edu.sa; 4 Botany and Microbiology Department, Faculty of Science, Helwan University, Ain Helwan, Cairo 11795, Egypt

**Keywords:** angiogenesis, anti-inflammatory, anti-oxidative, diabetes, excision wound, incision wound

## Abstract

Green nanotechnology is now accepted as an environmentally friendly and cost-effective advance with various biomedical applications. The cyanobacterium *Synechocystis* sp. is a unicellular spherical cyanobacterium with photo- and hetero-trophic capabilities. This study investigates the ability of this cyanobacterial species to produce silver nanoparticles (AgNPs) and the wound-healing properties of the produced nanoparticles in diabetic animals. Methods: UV–visible and FT-IR spectroscopy and and electron microscopy techniques investigated AgNPs’ producibility by *Synechocystis* sp. when supplemented with silver ion source. The produced AgNPs were evaluated for their antimicrobial, anti-oxidative, anti-inflammatory, and diabetic wound healing along with their angiogenesis potential. Results: The cyanobacterium biosynthesized spherical AgNPs with a diameter range of 10 to 35 nm. The produced AgNPs exhibited wound-healing properties verified with increased contraction percentage, tensile strength and hydroxyproline level in incision diabetic wounded animals. AgNPs treatment decreased epithelialization period, amplified the wound closure percentage, and elevated collagen, hydroxyproline and hexosamine contents, which improved angiogenesis factors’ contents (HIF-1α, TGF-β1 and VEGF) in excision wound models. AgNPs intensified catalase (CAT), superoxide dismutase (SOD), and glutathione peroxidase (GPx) activities, and glutathione (GSH) and nitric oxide content and reduced malondialdehyde (MDA) level. IL-1β, IL-6, TNF-α, and NF-κB (the inflammatory mediators) were decreased with AgNPs’ topical application. Conclusion: Biosynthesized AgNPs via *Synechocystis* sp. exhibited antimicrobial, anti-oxidative, anti-inflammatory, and angiogenesis promoting effects in diabetic wounded animals.

## 1. Introduction

Wound healing is a complicated multi-step process comprising clotting, inflammation, proliferative and remodeling stages that involves immunological, biochemical, and physiological communications [1,2]. Diabetes mellitus (DM) is a disorder that involves several systems and causes the wound healing process to be impaired. This in turn would trigger a delayed healing or chronic ulcers of the skin. This may lead to increased infections, poor contraction of the wound and decreased breaking strength of the wound [3]. The mechanisms behind delayed healing of the diabetic wound are complicated [4]. Dysfunction of wound healing disables injured tissues’ capability of healing and regeneration [5]. Delayed healing may be attributed to an inflammatory response imbalance, changed cytokines production, and collagen synthesis. This subsequently can result in inadequate angiogenesis, as well as reduced tensile strength and growth factors [6].

During the proliferative stage of healing, angiogenesis occurs where major angiogenic factors such as vascular endothelial growth factor (VEGF) and transforming growth factor (TGF)-β1 are involved in the stimulation, promotion, and stabilization of new blood vessels [5,7]. Moreover, hypoxia in wound injury stabilizes hypoxia-inducible factor-1α (HIF-1α), which mediates the synthesis of VEGF, stromal cell-derived factor (SDF-1α), and heme oxygenase-1 (HO-1), involved in neovascularization, via the transactivation of the respective genes, which is vital for wound healing [8]. Following hypoxia, several proangiogenic factors such as VEGF stimulate capillaries to form nascent immature loops and branches [9]. 

Diabetic wounds exhibit inadequate angiogenesis with significant perturbations in the expression of factors that affect vascular regrowth, maturation, and stability. More specifically, the expression of VEGF-A, HB-EGF, EGFR, TGF-β1, Neuropilin 1, angiopoietin 2, SDF-1α, HIF-1α, and RGS5 were all downregulated in diabetic wounds [7]. A reduction in the HIF-1α level results in decreased expression of VEGF and SDF1α, which leads to aberrant neovascularization, and impaired wound healing in diabetes [10]. 

In diabetic wounds, continuous inflammation promotes the recruitment of neutrophils that release various cytokines, causing extensive tissue damage to peripheral cells [2]. The continuous influx of neutrophils, excess of pro-inflammatory cytokine such as tumor necrosis factor alpha (TNF-α), and downregulation of the anti-inflammatory cytokine interleukin-10 (IL-10) all together lead to the generation of additional free radicals with increased protease activity, and consequently, persistent inflammation in diabetic wounds [11]. Chronic inflammation with the subsequent failure of fibroblast growth and collagen synthesis work together to delay the healing processes [12]. 

Furthermore, hyperglycemia results in the overproduction of reactive oxygen species, causing further complications and delayed wound healing in diabetic patients [13]. The extensive tissue injuries become beyond repair in diabetic lesions as they are accompanied by the increased production of free radicals, there by causing oxidative stress that leads to harmful cytotoxic products, which in turn causes delayed wound healing. Therefore, oxidative stress and inflammation perform a major role in the delayed recovery of wounds in diabetic patients. From the above-mentioned facts, it is apparent that elements that possess both antioxidant and anti-inflammatory impacts may decrease the level of oxidation and inflammation in diabetic rats, thereby accelerating wound healing. 

Conventional therapies for wound healing are, somehow, incompetent due to changes in the molecular constituency of the injured area. Thus, there is a need for effective wound treatment. In that regard, nanoparticles are known to promote healing of wounds by facilitating proper transitions through the healing phases [14]. Nanoparticles (NPs) have good functionality, biocompatibility, and ability to target specific cells; thus, they are more likely to gain access into cytoplasm or nuclei due to their significantly larger surface-area-to-volume ratio. As a result, they offer an augmented response as well as different plausible physiochemical and biomedical characteristics from bigger particles [15]. NP production by physical or chemical synthetic methods needs expensive apparatus, reagents, and toxic solvents which lead to hazardous by-products, causing contamination to both the environment and biosystems [16]. NP green synthesis is quickly developing as a key branch of nanotechnology where NPs are produced via biological entities such as plant extracts [17,18] and microorganisms [19,20] in an eco-friendly manner. Among the different inorganic metals in use as nanoparticles is gold, copper, iron, titanium, and zinc. Nevertheless, silver is the most prevalent metal in therapeutic applications owing to its inherent antimicrobial properties. Silver nanoparticles (AgNPs) showed numerous pharmacological actions including anticancer [21], anti-inflammatory [14], antioxidant [22], and anti-diabetic [14]. Furthermore, AgNPs mitigated hepatic injury induced by murine blood-stage malaria infection [23], and cisplatin-induced nephrotoxicity [17]. 

*Synechocystis* sp. is a unicellular cyanobacterial species belonging to the family Merismopediacea. It is photosynthetic but not diazotrophic, unlike other species of cyanobacteria such as *Cyanothece* sp. Cyanobacteria are the center of extensive studies as models for genetic manipulation, transcriptomic and proteomic studies, as well as some metabolomics and secondary metabolites synthesis investigations [24]. 

Therefore, the present study investigates the biosynthesis and characterization of AgNPs produced by the local cyanobacterium *Synechocystis* sp. The wound healing effect of the biosynthesised AgNPs on excision and incision and diabetic wounds was examined through identifying the anti-inflammatory, antioxidant, and angiogenesis actions.

## 2. Results

### 2.1. Characterization of AgNPs Biosynthesized by Synechocystis *sp.*

**Figure 1:**
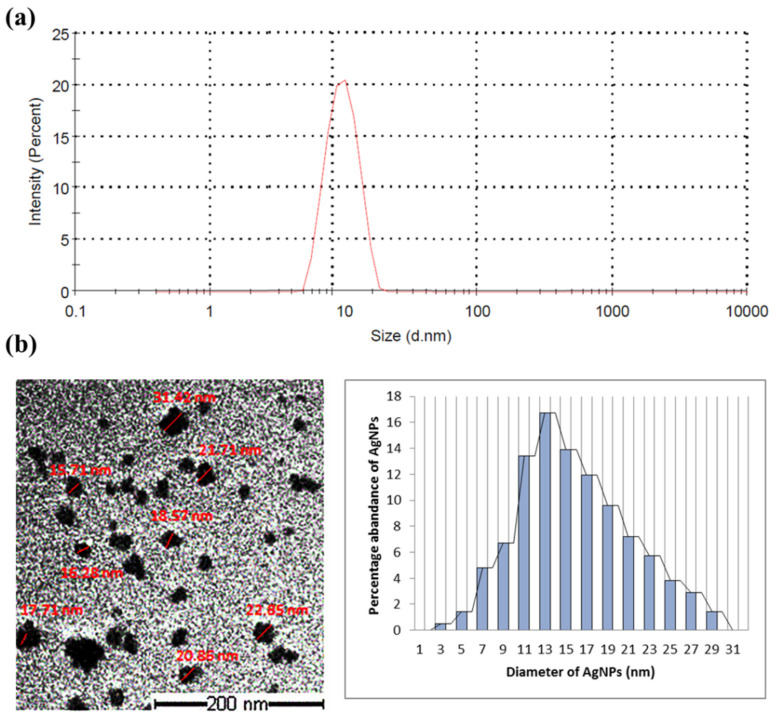
Particle size measuring of the AgNPs synthesized by *Synechocystis* sp. (**a**) Dynamic scattering graph identifying the particle size; (**b**) TEM images of the AgNPs with measured diameter (in red) and histogram of size distribution.

The bioproduction of AgNPs by *Synechocystis* sp. was preliminarily indicated by the change in the reaction mixture color, containing the cyanobacterial cell and AgNO_3_, when compared to the negative control. The transit color change from yellowish green to brownish suggests the reduction Ag^+^ ion to Ag^0^ as well as the development of AgNPs. A dynamic light scattering technique was used to measure the particle size of the produced AgNPs, found to be 7–35 nm (Figure 1a). TEM imaging showed that the particles were spherical in shape with a diameter ranging from 10 to 35 nm, the average particle size being around 16 nm and the most abundant nanoparticles being those of a diameter of 13 nm (Figure 1b).

**Figure 2:**
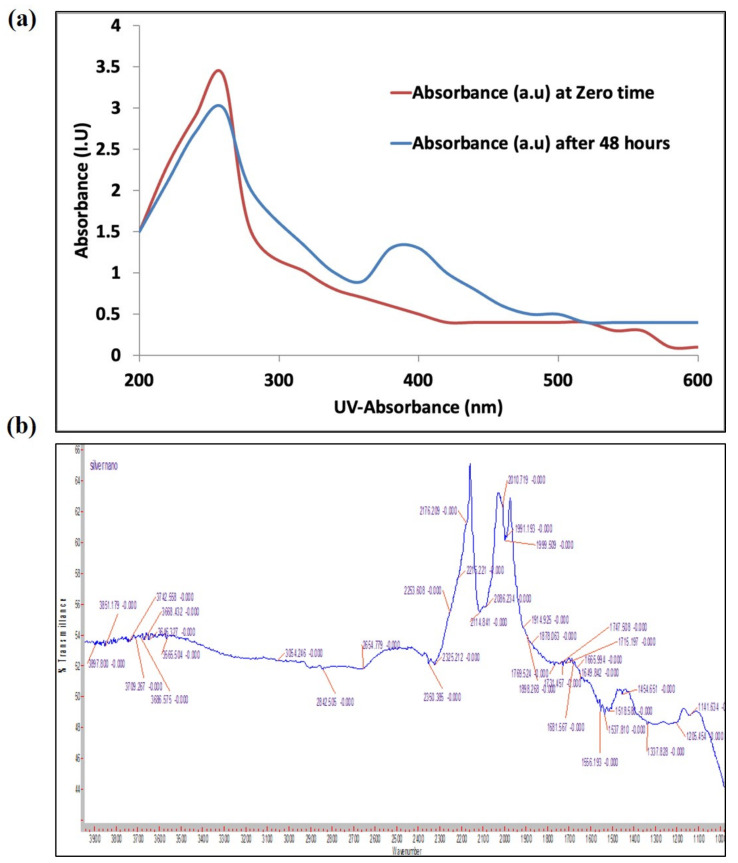
Characterization of biogenic AgNPs synthesized by *Synechocystis* sp. (**a**) UV–Vis light spectroscopy at 0 and 48 h of addition of AgNO_3_ to the cyanobacterial culture; (**b**) FTIR spectrum of cyanobacterial extract after 48 h of AgNO_3_ addition.

The frequency and width of the surface plasmon absorbance depends largely on the size and shape of the metal nanoparticles as well as on the dielectric constant of the metal and the surrounding medium. The biosynthesized AgNPs gave a broad surface plasmon absorbance at 380–420 nm, indicating the presence of AgNPs [25]. The broadening of the peak indicated that the synthesized AgNPs are polydispersed in nature (Figure 2a). There were signs of aggregation with the biosynthesized AgNPs solution. The diameter of nanoparticles produced in our study is around 13–15 nm. This small size particle gave the nanoparticles high surface energy, so the particles tend to agglomerate to diminish this energy. Furthermore, we think that the cyanobacterial solution did not provide enough stabilization to these small-sized nanoparticles, and this is another reason for them to agglomerate, in order to minimize the interface. 

An FT-IR spectrophotometer (Figure 2b) was used to identify the absorption spectra of the cyanobacterial species alone and with AgNPs complexes. The spectral absorbance bands of the nanoparticles were seen in the range of 3668–3054 cm^−1^ (H—bonded alcohols, phenols), 2842 cm^−1^ (CH_2_, alkanes), 2654 cm^−1^ (S-H stretch, thiol), 1649 cm^−1^ (N-H bend, primary amines), and 1114 cm^−1^ (C-N stretch, aliphatic amines). 

### 2.2. Antimicrobial Assay of AgNPs Produced by Synechocystis *sp.*

**Figure 3:**
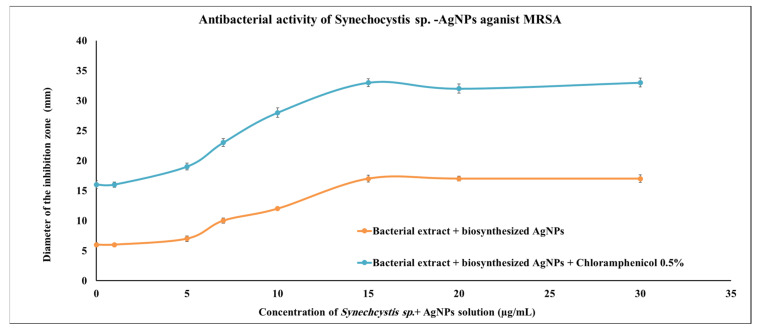
Antimicrobial activities of *Synechocystis* sp. extract with the biosynthesized AgNPs. Scatter plot of disc inhibition zone (mm) of *Synechocystis* sp. extract + AgNPs against MRSA with and without 0.5% chloramphenicol.

AgNPs produced by *Synechocystis* sp. were assessed for their anti-MRSA activity using the disc-diffusion method. Several concentrations of the cyanobacterial-AgNP extracts were investigated, and the lowest concentration with significant action was 10 μg/mL; however, the optimum concentration was 15 μg/mL, which was able to produce nearly the same activity as the positive control (0.5% chloramphenicol). When the same concentration (15 μg/mL) of *Synechocystis* sp.-AgNPs extract was combined with 0.5% chloramphenicol, the antibacterial activity increased nearly to 100% more than the 0.5% chloramphenicol alone (Figure 3).

### 2.3. Incision Diabetic Wound Healing Evaluation of AgNPs Produced by Synechocystis *sp.*

The wound contraction percentage of incision wounds created in the diabetic rats treated with the 10 μg/kg AgNPs and 30 μg/kg AgNPs were statistically (*p* < 0.05) different from those in the diabetic negative and diabetic positive control groups. The diabetic positive control rats were treated with the commercial standard ointment (Silver Sulfadiazine 1% ointment (SSD) (Dermazin^®^). The wound contraction percentage after treatment with the AgNPs ointments provided a significant improvement, reaching 85.69 ± 4.5% and 89.4 ± 6.32% on the 14th day, when related to diabetic negative control (50.96 ± 3.91%). The healing ratio exhibited a moderate improvement when compared to the diabetic positive control (74.7 ± 5.4%), as presented in Table 1. However, there was no significant difference between the two concentrations 10 and 30 μg/kg AgNPs in wound contraction percentage. On day 21, the diabetic positive control and AgNPs-treated animal reached full closure of the wound (100%) while the negative control, which did not receive any treatment, showed delayed wound closure (88.49 ± 12.27%).

The tensile strength and hydroxyproline level for each group in the experiment were measured and reported in Table 1. The breaking strength was found to be highest in the 30 μg AgNPs/kg followed by 10 μg AgNPs/kg groups, positive-diabetic, and finally, the negative-diabetic control groups, which showed the lowest tensile strength. Furthermore, there was a significant decrease in hydroxyproline content in negative diabetic wound animals as compared to positive diabetic wound animals. The 10 and 30 μg AgNPs/kg treated dietetic animals showed a significant (*p* < 0.05) increase in the levels of hydroxyproline when compared to both diabetic positive and negative controls, indicating enhanced healing processes.

### 2.4. Excision Diabetic Wound Healing Evaluation of AgNPs Produced by Synechocystis *sp.*

**Figure 4:**
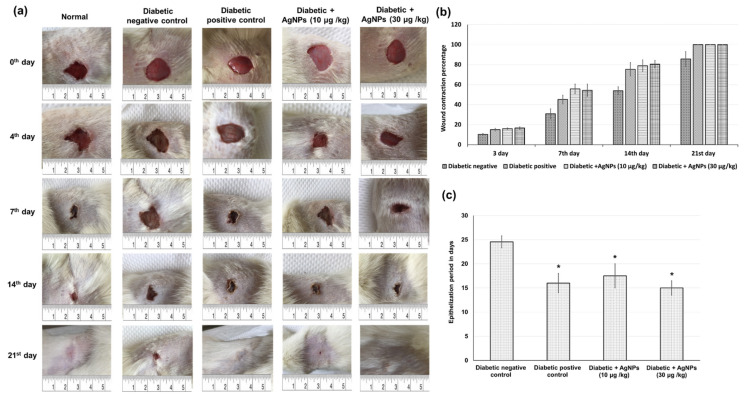
Effect of treatment with AgNPs green-synthesized by *Synechocystis* sp. on wound healing parameters including (**a**) photographic illustration of wound healing progression (**b**) wound contraction percentage in excision wound model obtained from different experimental groups at 0, 3, 7, 14, and 21 days of post wounding (**c**) epithelialization period. All values were expressed as mean ± SD (*n* = 6). * indicates statistically significant from diabetic negative control group (*p* < 0.05) using one-way ANOVA followed by Tukey’s test as a post hoc analysis.

Quantitative measurements of wound size are employed to assess initial wound size before and after debridement, as well as wound closure progression. Photographs of the wound and wound area measurement were performed at the day of wound creation, that is, 0 and on days 3, 7, 14, and 21 post-wound creation. Figure 4 shows illustrative wound shots taken on different days for each experimental group to evaluate the healing potential of AgNPs in diabetic wounds. Initially, a bright red color was observed in all wounds on the first day after the wound creation, signifying that the blood was recovered to the underlying muscle after the skin injury. On the seventh day after wound creation, a dark brown color was detected for the AgNPs-treated groups and diabetic positive control, signifying scab formation, while the untreated diabetic negative wounds were still slightly red and inflamed. On the 14th day after wound creation, AgNPs-treated wounds showed a significantly reduced wound size as compared to untreated diabetic negative animals, as demonstrated in Figure 4. After 21 days of wound injury, the untreated diabetic rats still showed an open wound (about 14%), in contrast to the treated groups in which a total contraction of wounds was achieved. The mean period of epithelialization increased in the diabetic negative control group, while it decreased in the AgNPs-treated groups and diabetic positive control group with no significant differences between the different treatment groups (Figure 4). The wound contraction percentage of excision wounds created in the diabetic rats treated with the 10 and 30 μg/kg AgNPs were statistically (*p* < 0.05) increased when related to the diabetic positive control group. However, there was no significant difference between the two concentrations 10 μg/kg AgNPs and 30 μg/kg AgNPs and positive control animals in the excision wound contraction percentage. The period of epithelialization was significantly increased in the positive control and AgNPs-treated animals when compared to negative-control rats.

**Figure 5:**
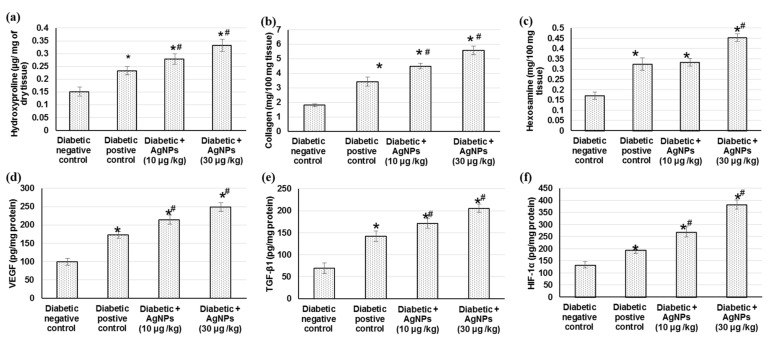
Effect of treatment with AgNPs green synthesized by *Synechocystis* sp. on (**a**) hydroxyproline content, (**b**) collagen, (**c**) hexosamine, (**d**) VEGF, (**e**) TGF-β1, and (**f**) HIF-1α in excision wound model. All values are expressed as mean ± SD (*n* = 6). * indicates statistically significant from diabetic negative control group, # indicates statistically significant from diabetic positive control group (*p* < 0.05) using one-way ANOVA followed by Tukey’s test as a post hoc analysis.

Collagen is the principal extracellular protein in the skin tissue, from which hydroxyproline is the main element, which makes it an excellent biochemical marker for collagen content within the tissue. Therefore, hydroxyproline amount variations can indicate any alteration in collagen synthesis, which reflect the process of wound healing in the damaged tissues. The diabetic negative control group exhibited lowered amounts of collagen, hydroxyproline, and hexosamine. As demonstrated in Figure 5, a significant increase in hydroxyproline levels was observed in AgNPs-treated groups (0.27 ± 0.019, and 0.33 ± 0.023 µg/mg of tissue), respectively, when compared to the negative untreated control (0.15 ± 0.017 µg/mg of tissue) and positive treated groups (0.23 ± 0.016 µg/mg), indicating that AgNPs induced hydroxyproline production. Accordingly, AgNPs-treated animals showed significantly (*p* < 0.05) increased levels of collagen and hexosamine, demonstrating improved healing processes, as shown in Figure 5. 

#### 2.4.1. Effect of AgNPs Biosynthesized by *Synechocystis* sp. on Angiogenesis Related Factors

Angiogenesis during wound repair serves a dual function of providing the nutrients required by the healing tissue and serving for structural repair through the formation of granulation tissue [9]. The results from the current investigation showed that the angiogenesis-related factors including HIF-1α, TGF-β1, and VEGF contents in wound tissues were lower in the negative diabetic animals than those from positive diabetic animals. Furthermore, AgNPs-treated animals exhibited a significant increase in HIF-1α, TGF-β1, and VEGF levels compared with tissue specimens obtained from rats in the diabetic positive group (Figure 5). Animals treated with 30 µg/kg of AgNPs exhibited significant higher levels of these angiogenesis factors compared to those treated with 10 µg/kg.

#### 2.4.2. Effect of AgNPs Biosynthesized by *Synechocystis* sp. on Antioxidant Enzymes Activities, Lipid Peroxidation and Nitric Oxide

**Figure 6:**
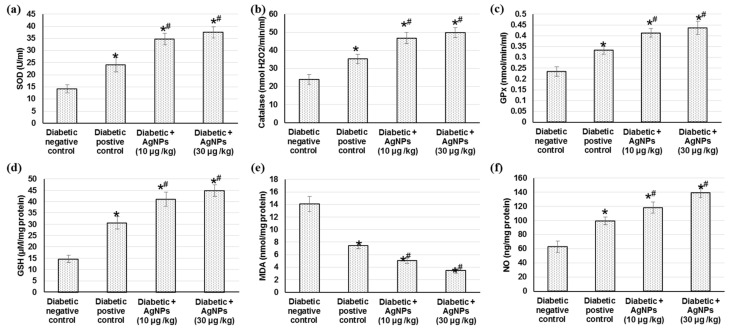
Effect of treatment with AgNPs green-synthesized by *Synechocystis* sp. on (**a**) SOD; (**b**) catalase; (**c**) GPx; (**d**) GSH; (**e**) MDA and (**f**) NO in excision wound model. All values were expressed as mean ± SD (*n* = 6). * indicates statistically significant from diabetic negative control group, # indicates statistically significant from diabetic positive control group (*p* < 0.05) using one-way ANOVA followed by Tukey’s test as a post hoc analysis.

Oxidative stress persistence during wound healing is detrimental, particularly in diabetic wounds. Figure 6 shows the outcomes of treatment with 10 and 30 µg AgNPs green-synthesized via *Synechocystis* sp. on different antioxidant enzymes such as catalase (CAT), superoxide dismutase (SOD), catalase (CAT), glutathione peroxidase (GPx), on the glutathione (GSH) content, lipid peroxidation such as malondialdehyde (MDA) and finally on the NO content on the excision wound healing process. Initially, there was a significant decrease in antioxidant enzymes including CAT, SOD, and GPx and GSH content and nitric oxide levels as well as increased lipid peroxidation in diabetic non-treated wound control when compared with diabetic positive animals (*p* < 0.05). The current study presented a significant elevation in antioxidant enzymes (CAT, SOD, and GPx) and GSH content in animals treated with AgNPs, as compared to diabetic positive animals, which could be attributed to the decrease in reactive oxygen species (ROS) production with AgNPs treatment. However, no significant difference was found between the two different doses of AgNPs 10 and 30 µg/kg. In addition, a notable reduction in MDA level (as a lipid peroxidation marker) in the tissue obtained from animals treated with AgNPs as compared to the positive control group tissue, indicating that the use of AgNPs reduced the secondary oxidation product content. Furthermore, the administration of AgNPs significantly increased the nitric oxide level when compared to diabetic positive control rats. 

#### 2.4.3. Effect of AgNPs Biosynthesized by *Synechocystis* sp. on the Inflammatory Mediators

**Figure 7:**
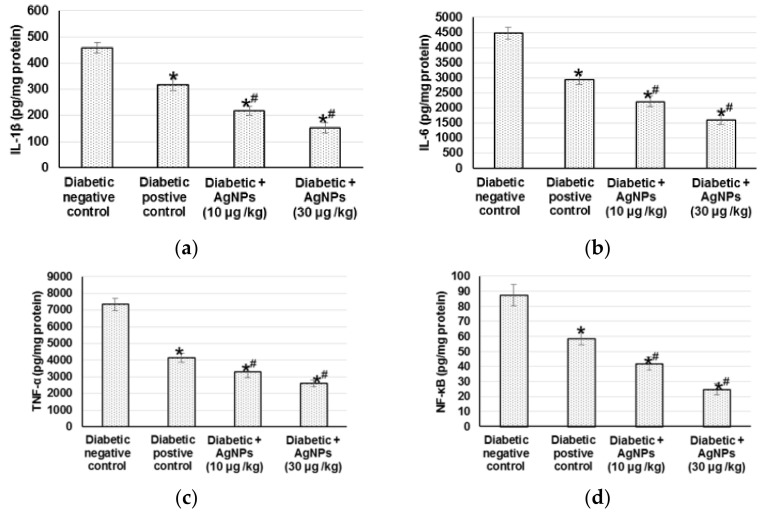
Effect of treatment with AgNPs green synthesized by *Synechocystis* sp. on (**a**) IL-1β; (**b**) IL-6; (**c**) TNF- and (**d**) NF-κB in excision wound model. All values were expressed as mean ± SD (*n* = 6). * indicates statistically significant from diabetic negative control group, # indicates statistically significant from diabetic positive control group (*p* < 0.05) using one-way ANOVA followed by Tukey’s test as a post hoc analysis.

The sustained oxidative stress can activate various transcription factors including NF-κB, which lead to chronic inflammation. Therefore, we measured different inflammatory mediators to indicate the inflammation status occurring within diabetic wound healing. Diabetic non-treated animals demonstrated significant increase in different inflammatory mediators such as IL-1β, IL-6, TNF-α, and NF-κB when related to positive diabetic control. Animals treated with 10 µg/kg and 30 µg/kg AgNPs exhibited lowered inflammation, as evidenced by the significantly lower level of inflammatory mediators when compared to diabetic positive animals (Figure 7).

## 3. Discussion

### 3.1. Characterization of AgNPs Biosynthesized by Synechocystis *sp.*

The use of *Synechocystis* sp. for the biosynthesis of nano-metals was reported once by Fathy et al. [26], who used an extract of *Synechocystis* sp. for the in vitro synthesis of AgNPs and used the synthesized nanoparticle as a flocculent agent with different microalgae strains [26]. The cyanobacterium *Synechocystis* sp. are rich in entities/bioactive compounds, which have reducing power for ions within their vicinity turning them into ecofriendly nanoparticles [27,28]. For instance, *Synechocystis* sp. PCC 6803 stain presents ascorbic acid and gluthathione, which are the two key components of the antioxidant machinery of eukaryotic and prokaryotic cells [29]. In the current study, the UV spectrum showed a stretching of plasmon resonance within 370–410 nm, which is characteristic of nanosilver [21]. The FTIR results exposed that the compounds that present in the cyanobacteria are most likely alkanes, amides, or amines, which could be responsible for the bioreduction of Ag^+^ and capping/stabilization of AgNPs. The FTIR spectrum indicated the presence of OH groups (3500 to 3200 cm^−1^, weak, broad bands for O-H stretching vibration). These OH groups can be from phenols, alcohols, or carboxylic acids. OH groups could be involved in nanoparticles biosynthesis [30]. Additionally, the FTIR spectrum shows the presence of SH (thiol) groups (2654 cm^−1^, medium, sharp band for S-H stretching vibration). SH groups showed potential activity as reducing agents and could contribute to the biosynthesis and stabilization of the AgNPs [31]. The band at 2842 cm^−1^ (weak) could be assigned to stretching vibrations of skeletal CH_2_ in polysaccharides [32] or fatty acids [33]. Both polysaccharides and fatty acids could play a role in the biosynthesis or stabilization of AgNPs [34,35,36]. The presence of CH_2_ for polysaccharides or fatty acids was further emphasized by the presence of a weak band at 1454 cm^−1^ (CH_2_ bending vibration) [33]. The presence of proteins or polypeptides is evidenced from the presence of bands at 1649 cm^−1^ (N-H bending vibration, primary amines or amides I), 1450 cm^−1^ (N–H bending vibration amide II), and 1114 cm^−1^ (C-N stretching vibration, aliphatic amines) [37,38]. Function groups in polypeptides and proteins could contribute to the biosynthesis or stabilization of AgNPs [39,40]. Those functional groups may act as capping agents of silver nanoparticles, resulting in the stabilization of AgNPs, which is in agreement with [20,41].

### 3.2. AgNPs Anti-Microbial Properties

AgNPs have two distinct anti-microbial impacts: inhibitory action and bactericidal action. AgNPs interacts with the proteins’ thiol groups in the bacterial cell, forming disulfide bonds, which interfere with many biological processes of bacterial cells [42], such as DNA replication and inhibit the bacterial growth leading to apoptosis [43]. On the other hand, the AgNPs bactericidal effect was established due to the ability of silver to create free radicals, causing bacterial cell wall damage. The bacterial cell then liberates lipopolysaccharides and membrane protein, resulting in cell mortality. Moreover, AgNPs inhibited the protein substrate phosphorylation, affecting signal transduction as verified earlier [44]. Indeed, in this study, green bioproduced AgNPs were able to act on MRSA with an activity similar to that of chloramphenicol, and the nanoparticle synergistic activity with chloramphenicol increased the antibacterial activity of the broad-spectrum antibiotic nearly by double.

### 3.3. AgNPs Diabetic Wound Healing Properties

There are four models to study wound healing: in silico, in vitro, ex vivo, and in vivo models. Direct analysis of a stimulus in the living subject before/during/after exposure to a stimulus is provided in the in vivo study, and that is why we have chosen this model. Many wound healing models have employed rats because of their wide availability, size, and tractable nature. Rats are large enough to provide a suitable area of skin for wound studies. Another reason to consider rats as skin wound healing models is the availability of a broad knowledge base on rat wound healing gained from years of previous research. Characteristics such as a short gestation, short lifespan, docile behavior, and ready availability of animals with well-defined health and genetic backgrounds are key in the decision to use the rat as the choice of a research animal [45]. Wound healing in rats is governed by myofibroblast-mediated contraction through an extensive subcutaneous striated muscle layer called the panniculus carnosus, which is absent in humans [46], and this represents a disadvantage in the rat wound-healing model. However, developing an animal model that has all the complexity of human chronic wounds may be an unattainable goal, because non-healing and delayed healing wounds in humans are often the result of combinations of impaired circulation, inadequate nutrition, age, limited physical activity, and/or chronic physiological imbalance. With the continuing developments in genetic manipulation of rats and other animal kinds, innovative, more beneficial models of the wound healing models will ultimately appear [46]. The worldwide prevalence of diabetes has increased strikingly, which has attracted the attention of both the scientific and public communities. The wound healing process relates to imperative phases, including the inflammatory reaction, cellular relocation, propagation, matrix deposition, and tissue restoration [4]. Many modulators such as cytokines, growth factors, matrix metalloproteinases (MMPs), cellular receptors, and extracellular components are associated with the wound healing process. The impaired wound healing may propagate to gangrene and amputation [47]. Delayed diabetic wound healing is mainly attributable to inflammatory response disproportion, modified cytokines production, changed collagen synthesis, inadequate angiogenesis, and declined growth factors [6]. Several studies have reported that nanoparticles can stimulate tissue healing and restoration in wound spots. Nanoparticles with dimensions less than 100 nm have a higher surface-area-to-volume ratio [48] and, consequently, higher admission into cytoplasm or nuclei to alter molecular processes. As a result, nanoparticles exhibit an augmented reaction with their physiochemical and biomedical properties that are different from other bigger particles [49]. AgNPs have been reported to possess wound-healing properties [50,51]. However, the enhancing effects of AgNPs on wound healing in diabetes have been poorly studied. In this study, we demonstrated that bio-synthesized AgNPs could improve the healing of both excisional and incisional wounds in STZ-induced diabetic rats. The green synthesis provides non-toxic AgNPs with well-defined size, which explains the recent research interest in those particles.

A critical stage of the wound repairing process is re-epithelialization, during which keratinocytes are permitted to transfer across the wound bed for epidermal layer re-establishment. Re-epithelialization aids in wound closing by switching keratinocytes to the migratory and proliferative phenotype, which is usually impaired in diabetes [52]. Our results showed prolonged wound closure in diabetic wounded rats accompanied by an increase in epithelialization time. These results were supported by earlier studies, which reported that hyperglycemia impairs keratinocytes migration and proliferation, creating insufficient re-epithelialization and longer epithelialization duration [4]. On the other hand, an increase in the wound closure percentage as well as a reduction in the epithelialization time in the diabetic wound rats treated with AgNPs was observed. This wound behavior is in accordance with former outcomes that AgNPs cause quicker wound closure and re-epithelialization of the epidermis at the wound spot in nondiabetic animals [29]. 

The healing progression majorly depends on the controlled manufacture and deposition of new collagen. Collagen is a central constituent of the extracellular matrix, and its production, placement, remodeling, and maturation are imperative stages throughout tissue restoration and regeneration [53], eventually contributing to wound tensile strength [54]. Thus, the wound strength is developed from both collagen transformation and the construction of stable intra- and intermolecular cross-links. Collagen offers strength and integrity to the tissue matrix, as well as playing a curial role in homeostasis and epithelialization of the healing process [55]. Collagen metabolism releases free hydroxyproline and its peptides; therefore, the extent of the hydroxyproline can be used as a collagen metabolism index [56]. Additionally, amplified hexosamine amounts stabilize the collagen molecules by accelerating electrostatic and ionic interactions [55]. Adequate wound healing requires hexosamine as a component of the ground substance for extracellular matrix synthesis [56]. Hexosamine intensification indicates that fibroblasts actively synthesize, on which collagen is laid down [57]. 

In the current experiment, investigational diabetic rats displayed decreased collagen, hydroxyproline, and hexosamine contents, which was revealed as very little or no formation of granulation tissue on diabetic wounds, as mentioned earlier [4,56]. AgNPs augment hydroxyproline and the hexosamine content with subsequent amplified collagen synthesis and deposition, increased granulation tissue formation, and improved extracellular matrix synthesis, and thus, proper wound healing. AgNPs are believed to improve collagen deposition during wound healing [14,42].

### 3.4. Angiogenesis Related Factors

When tissue injury occurs, the normal homeostasis is disturbed, leading to a hypoxic state. Hypoxia activates hypoxia-inducible factor-1 (HIF-1) which is a transcriptional activator that intensifies angiogenesis by upregulating specific genes such vascular endothelial growth factor (VEGF) and transforming growth factor β1 (TGF-β1) [58]. VEGF is a proangiogenic mediator which stimulates endothelial cell functions necessary for the formation of new blood vessels. VEGF encourages angiogenesis by stimulating endothelial cell propagation and averts their apoptosis as well as guides vascular growth to low-oxygen zones, starting from the wound borders to the bed [59]. Therefore, VEGF plays a crucial part in tissue propagation, relocation, and differentiation, contributing to angiogenesis and influencing wound repair, closure, and granular tissue establishment [60]. Another important growth factor is TGF-β1, which plays a pivotal role in mediating collagen synthesis and degradation. During the wound healing process, TGF-β1 is secreted and is involved in numerous roles, such as inflammation, angiogenesis, collagen production and deposition, extracellular matrix remodeling [61], and fibroblasts migration and proliferation [62]. Thus, increasing the endogenous release of growth factors is a potential mechanism of wound tissue repair. 

In the present study, VEGF, TGF-β1, and HIF-1 were down-regulated in diabetic negative wounded rats when related to positive diabetic controls. Previously, diabetic patients were reported to have unusually lower levels of VEGF, which is concomitant with inadequate vascularization accompanied by wound closure complications and diminished granular tissue re-epithelialization and development [63]. On the contrary, topical administration of 10 µg/kg and 30 µg/kg AgNPs exhibited an upregulated VEGF level in diabetic wound rats, signifying the angiogenic potential of AgNPs. In addition, TGF-β1 and HIF-1 were also augmented in AgNPs-treated animals, indicting enhanced angiogenesis, re-epithelialization, and a faster rate of wound closure when related to diabetic negative controls. Similarly, AgNPs increased VEGF synthesis, stimulated angiogenesis, and facilitated wound healing in primary cell cultures of fibroblasts and keratinocytes in a wound-healing model [50]. 

### 3.5. Antioxidant Enzymes Activities

Oxidative stress and inflammatory states’ existence at the wound site is detrimental for healing progression, especially diabetic cases. Throughout the repair process, reactive oxygen species (ROS) are created when molecular oxygen is reduced by NADPH oxidases [64]. Cell migration and proliferation are hindered and the inflammation mediator’s expression and functions are affected by oxidative stress [65]. In the absence of appropriate antioxidant actions, the wound healing process might be delayed. Therefore, adequate wound restoration endorses various antioxidant genes such as glutathione peroxidase (GPx), catalase (CAT), and SOD [66]. The outcomes of the existing experiment demonstrated lipid peroxidation (MDA) level augmentation as well as antioxidant enzyme activity diminution including SOD, CAT, GPx, GST, and GR in diabetic wounds compared to normal wounds. The management of diabetic wound animals with both 10 and 30 µg/kg AgNPs significantly diminished the MDA levels and augmented antioxidant defenses activities linked to diabetic wound controls. The outcomes are similar to previous studies which revealed increased MDA levels and decreased antioxidant activities in the diabetic wound tissue [4]. ROS upsurges in diabetes mellitus due to glucose auto-oxidation, advanced glycation, and an unusual mitochondrial role [67], initiating granulation tissue damage along with diminished angiogenesis and the relocation of fibroblast and keratinocytes [68]. In a diabetic wound model, a high blood-glucose level prompted the formation of free radicals, lowered the antioxidant enzyme activities, and eventually delayed injury healing [69]. SOD and GPx serve as free radical scavengers. For instance, GPx neutralizes the hydrogen peroxide into water [70], while SOD dismutates superoxide radicals into less toxic hydrogen peroxide and dioxygen free radicals [71]. By expanding CAT and GPx enzyme actions, the resultant hydrogen peroxides were enzymatically neutralized into oxygen and water [71]. The explanation could be that in wounded diabetic rats, the Nrf2 mRNA expression was lowered with an upregulation in the Keap1 mRNA expression level [4]. Furthermore, in an STZ diabetic mouse model, Nrf2^−/−^ mice showed a deferred wound healing level in contrast to Nrf2^+/+^ mice, indicating the Nrf2 part in wound healing [72]. 

On the other hand, AgNPs’ management caused greater collagen placement, NO production, and enhanced endogenous antioxidants, which was reflected in condensed wound regions and better wound healing [56]. Our outcomes clearly advocate that AgNPs revealed a synergic influence of an effective antioxidant action by lowering the oxidative stress in the wound area. Additionally, the current study showed that treatment AgNPs significantly promoted NO levels in the diabetic wound fluid. Evolving suggestions specified that NO has a significant role in normal wound healing and that cutaneous NO dysfunction is consistently elaborated in wound healing impairment [73]. NO promotes numerous processes, including angiogenesis and relocation and propagation of fibroblasts, epithelial cells, endothelial cells, and keratinocytes [74]. Different cells in a wound have the capability to produce NO, including platelets, macrophages, fibroblasts, endothelial cells, and keratinocytes. Nitric oxide stimulates cell proliferation, angiogenesis, and regeneration [75]. 

### 3.6. Inflammation and Inflammatory Mediators

Throughout wound restorative progression in diabetic animals, the expression of NF-κB pathway genes is exaggerated to trigger the inflammatory cytokines (TNF-α, IL-1β, and IL-6) expression, causing deferred wound healing. The changes were effectively reversed in diabetic animals by treating 10 and 30 µg/kg AgNPs. Our results supported the hypothesis of AgNPs participation in the NF-κB and inflammatory response suppression to recover healing process. Continued oxidative stress may trigger several transcription factors including NF-κB, leading to prolonged inflammation. The NF-κB binding activity was significantly increased with an increase in TNFα, IL-1β, and IL-8 within the diabetic wounded skin homogenate [4]. Eming et al. [76] indicated that TNF-α and IL-1β elicited the inflammatory reaction by stimulating additional neutrophils and macrophages, which are indispensable for the regular repair process. TNF-α prevents SMAD phosphorylation via c-Jun N-terminal kinase pathway and diminishes TGF-β1 transcription [77]. Previously, it was hypothesized that the suppression of the triggered NF-kB pathway with the following subsequent inflammatory gene expression is intricate in the enhancement of wound healing process [78].

## 4. Materials and Methods

### 4.1. Cyanobacterial Strain

The cyanobacterium species *Synechocystis* sp. Was originally isolated from the Arabian Gulf coast, located in the eastern region of Saudi Arabia as mentioned previously [79]. The strain was isolated on BG11 medium and kept in a 12:12 light/dark cycle. The stain was morphologically identified using light microscopy.

### 4.2. Biosynthesis of Silver Nanoparticles AgNPs

Silver nitrate (AgNO_3_) used in the current study was obtained from Sigma-Aldrich (St. Louis, MO, USA). A stock solution of silver nitrate (AgNO_3_) of 0.5 mM concentration was prepared and kept in a dark bottle. An aliquot of 1 mL of silver nitrate (AgNO_3_) was added to a 19 mL culture of *Synechocystis* sp. And incubated at a temperature of 28 °C for 24 h. The same steps were repeated but with switching cyanobacterial biomass with distilled water to attain the negative control. The preliminary suggestion of AgNPs biosynthesis was visually observed when the solution color transformed to a brown color. 

### 4.3. Biosynthesized AgNPs Shape and Size Determination

The particle size of the biosynthesized AgNPs was characterized by dynamic light scattering techniques using Zetasizer Nano ZS (Malvern Panalytical, Spectris plc, Egham, Surrey, UK). Transmission electron microscopy (TEM, a JEOL JEM-1230 JEOL, Tokyo, Japan) was utilized to obtain high-resolution, two-dimensional images of the biosynthesized AgNPs. The AgNPs morphology was visualized via TEM operated at an accelerating voltage of 200 kV. Samples were prepared by adding a drop of AgNPs suspension onto a carbon-coated copper grid and permitted to dry under an infrared lamp preceding the analysis. Nanoparticle size distribution was performed using the digitized TEM images. Thirty haphazardly selected TEM fields were treated with Image Pro-Plus software and the data obtained were reported in a histogram (Figure 1). 

### 4.4. Characterization of the Produced Biosynthesis AgNPs

Characterization of AgNPs was accomplished using UV–Vis spectrophotometry (Genesys10S, Thermo Scientific, Waltham, MA, USA) in the range of 200–900 nm. In addition, to identify the functional groups involved in the bio-production AgNPs, Fourier-transform infrared spectrophotometer (FT-IR) was (Agilent Cory 630, Agilent Technologies, Austin, TX, USA) operated through an investigation region of 4000–500 cm^−1^. 

### 4.5. Preparation of AgNPs for Analysis

The cyanobacteria–AgNPs mixture was centrifuged (12,000× *g*, 20 min, 10 °C), and the generated pellets were washed with 10 mL distilled water numerous times. The obtained pellets were then dried (30 °C) for 24 h and spread in distilled water at the required concentrations. 

### 4.6. Antimicrobial Activity of Biosynthesis AgNPs

The antimicrobial effectiveness of AgNPs was evaluated against methicillin-resistant *Staphylococcus aureus* (MRSA) strain [27] using Kirby–Bauer disk diffusion susceptibility with minor amendment [80]. Different concentrations (including 1, 5, 7, 10, 15, 20, and 30 µg/mL) of AgNPs were prepared, in which sterilized Whatman number 1 paper discs (6 mm in diameter) were impregnated in 20 µL of AgNPs solution. The dried paper discs were positioned on a nutrient agar medium which was inoculated with the bacterial suspensions. To guarantee the diffusion of the AgNPs bioactive material, plates were maintained for 2 h at 4 °C and then incubated at 37 °C. Negative control discs contained 20 µL of sterilized distilled water, whereas the positive control discs enclosed 0.5% chloramphenicol. The AgNPs and chloramphenicol mixture was used where different concentrations of AgNPs were produced with 0.5% chloramphenicol solution. Sterilized-Whatman number 1 paper discs were impregnated in 20 µL of the prepared solution. The produced paper discs were dried and treated similarly to as mentioned before. All plates were incubated for 24 h, and the diameters of inhibition zones were measured in triplicates (mm) to be used as an indicator for activity.

### 4.7. AgNPs Diabetic Wound Healing Methods

#### 4.7.1. Animals

Male Wistar rats (250 ± 10 g) were obtained from the animal house facility, King Saud University, Riyadh, Kingdom of Saudi Arabia. The animals were retained in the animal house under typical surroundings (20–25 °C, 70–80% humidity, 12 h light/dark cycle) for a week preceding the experiment. They obtained standard laboratory chow and water ad libitum. Animals were haphazardly allocated into 6 animals per group. 

#### 4.7.2. Ethical Approval

All experiments were appropriately executed in agreement with the “Ethical Conduct for Use of Animals in Research” Guidelines in King Faisal University and the “Executive Regulations for Research Ethics on Living Creatures (Second Edition)”, published by the National Bioethics Committee, Saudi Arabia. All animal care and experimental procedures were approved by the Animal Research Ethics Committee at King Faisal University (KFU-REC-2021-NOV-EA000143).

#### 4.7.3. Induction of Diabetes

A recently prepared solution of streptozotocin (STZ) (Sigma, St. Louis, MO, USA) dissolved in citrate buffer pH 4.5 was administered to overnight-fasted rats at a dose of 65 mg/kg intraperitoneal (i.p.) to prompt diabetes. To avert hypoglycemia caused by the immense insulin discharge, animals were administered 10% glucose solution after 6 h of STZ injection for another 24 h [81,82]. After 72 h of the STZ administration, the blood was withdrawn via animals’ tail vein and the animals with a fasting blood glucose level (BGL) higher than 200 mg/dL were considered diabetic and employed in the current study.

#### 4.7.4. Grouping of Animals

Two wound models (excision and incision) were used to evaluate the diabetic wound healing activity in this investigation. Rats were arbitrarily separated into five groups in each model (*n* = 6). Group I: normal control group, in which normal rats were permitted to heal deprived of any treatment. Group II: diabetic negative control group, in which diabetic rats were granted to reconcile spontaneously without any treatment. Group III: diabetic positive control group, in which diabetic rats were treated with the commercial standard ointment (Silver Sulfadiazine 1% ointment (SSD) (Dermazin^®^), manufactured by Medical Union Pharmaceuticals, Saudi Arabia). Group IV: 10 µg/kg AgNPs-treated diabetic group, in which diabetic animals were treated with a topical application of 10 µg/kg AgNPs on wounds once daily. Finally, group V: 30 µg/kg AgNPs-treated diabetic group, in which diabetic rats were treated with topical application of 30 µg/kg AgNPs on wounds for once a day. Different concentrations of topical formulations (ointments) were prepared for all groups, including the negative control group, as mentioned elsewhere [22], and applied once daily, directly on the open wound until the wound was completely healed. Animals were monitored daily for any wound contamination. No antibiotic was given to any of the animals’ groups. At the end of the investigation and after the complete wound healing, the animals were sacrificed using 10 mg/kg of xylazine (Bayer, Leverkusen, Germany) and 25 mg/kg of ketamine (Pfizer Inc., New York, NY, USA) and tissue specimens were collected and maintained in liquid nitrogen for further biochemical analysis. At the end of the experiment, blood samples were collected, centrifuged at 3000× *g* for 15 min, and the resulting serum was utilized for numerous biochemical assays.

#### 4.7.5. Incision Wound Model Creation

This model was implemented as mentioned before in Süntar et al. [83] with minor modification. Briefly, an 8 cm straightforward incision was performed paravertebrally through the full skin thickness on both sides of the vertebral column with a sharp, sterile scalpel, then an interrupted suture (about 1 cm apart) was used to close the wound. Days of applications of topical preparations started from day 0 to the day of complete wound healing. Rats were randomly segregated into different groups as mentioned earlier. 

#### 4.7.6. Estimation of Tensile Strength

The tensile strength was employed as mentioned before in Yadav et al. [84]. At the end of the experiment, the skin area was incised 1.5 cm away from each side of the wound, aligned with the anterior–posterior axis of the animal to warrant sample consistency. Additionally, the thickness and width of the skin injury regions were evaluated using Vernier calipers. The acquired tissues were used to measure the force necessary to break the tissue using a computerized tensiometer (EZTESTI 30804100798, Shimadzu Corp., Kyoto, Japan). The tensile strength was calculated using the following formula:

Tensile strength (N/cm^2^) = Breaking force (N)/Area (cm^2^) where area (cm^2^) = thickness (cm) × width (cm)

#### 4.7.7. Excision Wound Model Creation

Anesthetized rats, via 10 mg/kg of xylazine and 25 mg/kg of ketamine i.p., were positioned face down on the dissection pad. Before the wound creation, the dorsal region of the anesthetized animals was shaved, and the wound areas were cleaned via 70% ethanol. An open circular cut of 0.2 mm depth and 10 mm^2^ diameter was made on the back of each animal using a sterile scalpel. The animals were then distributed randomly into different groups as mentioned earlier. Days of application of topical preparations started from day 0 to the day of complete wound healing.

#### 4.7.8. Measurement of Wound Area Parameters

Macroscopic investigation: Wound photographs were taken on day 0, 3, 7, 14, and 21 using a camera (Spot Insight QE; Diagnostic Instruments, Sterling Heights, MI, USA). The epithelialization time is defined as the number of days taken to drop off the dead tissue without any sign of raw wound, as formerly stated in [4]. The wound area was measured by marking tracing paper by placing the paper on the wound on the 0th, 7th, 14th, and 21st day after wound establishment. The percentage of wound contraction was calculated as stated previously [5] using the below equation:

Wound contraction percentage = ((wound area day 0 − wound area day n)/wound area day 0) × 100 where n represents the number of days until complete healing

#### 4.7.9. Skin Tissues Measured Parameters

Hydroxyproline content: the hydroxyproline content in wound tissues was assessed as described earlier [51,85]. Briefly, the wound tissues from different experimental animals were dried in a hot air oven at 60 °C to obtain similar weights and then hydrolyzed with 6 N hydrochloric acid (HCl) (1:10, *w*:*v*) at 130 °C for 4 h in sealed glass tubes. The attained hydrolysates were neutralized to pH 7.0 and subjected to oxidation via chloramine T. The reactions accomplished by the addition of perchloric acid (0.4 M), and Ehrlich reagent at 60 °C to develop the colors which were valued at 557 nm. A standard linear curve was preformed, from which hydroxyproline concentrations were calculated and presented as µg/mg of dry tissue weight.

To evaluate the collagen and hexosamine, firstly, the wound tissue samples were defatted in chloroform:methanol (2:1 *v*/*v*) and dried in acetone. Weighed tissues were hydrolyzed in 6 N HCl for 18 h at 110 °C, evaporated to dryness, and then made up with a known volume of water as described earlier [86,87]. 

#### 4.7.10. Measurement of Angiogenesis Related Factors

Vascular endothelial growth factor (VEGF, Item No. LS-F5482), transforming growth factor β1 (TGF-β1, Item No. LS-F24972) and hypoxia-inducible factor 1-alpha (HIF-1α, Item No. LS-F4225) levels in the tissue homogenates were estimated using the ELISA kit obtained from LifeSpan BioSciences, Seattle, WA, USA following the manufactures’ procedures. 

#### 4.7.11. Measurement of Antioxidant Enzymes Activities

Catalase (CAT, Item No. 707002), superoxide dismutase (SOD, Item No. 706002), glutathione peroxidase (GPx, Item No. 703102), and glutathione (GSH, Item No. 703002) were measured according to the manufacturer’s protocols using the corresponding ELISA kits, which were obtained from Cayman Chemicals (Ann Arbor, MI, USA). 

#### 4.7.12. Measurement of Lipids Peroxidation

The lipid peroxidation was determined by thiobarbituric acid reaction and expressed as the malondialdehyde (MDA) level using an MDA kit (Cat. No. ab 118790), which was obtained from Abcam (Cambridge, MA, USA).

#### 4.7.13. Estimation of Nitric Oxide (NO)

The stable end products of nitric oxide (NO) biosynthesis were estimated by appraising the nitrite levels. Greiss reagent (500 μL; 1:1 solution of 1% sulphanilamide in 5% phosphoric acid and 0·1% napthaylamine diamine dihydrochloric acid in water) was placed with 100 μL of serum and absorbance was valued at 546 nm using a double-beam UV–visible spectrophotometer [88]. The nitrite concentration was calculated using a standard curve for sodium nitrite and expressed as nanograms per milligram of protein.

#### 4.7.14. Measurement of the Inflammatory Cytokine

Tumor Necrosis Factor-alpha (TNF-α, Item No. LS-F23150), Interleukin 8 (IL-8, Item No. LS-F9753) and Interleukin 1β levels by ELISA based kits obtained from LifeSpan BioSciences, Seattle, WA, USA. The assays were preformed as per the manufacturer’s guidelines.

### 4.8. Statistical Analysis

Data are shown as the mean ± SD. Multiple comparisons were preformed via one-way ANOVA, followed by Tukey’s test for post hoc analysis, using a 0.05 level of probability as the significance level. All statistical analyses were achieved using GraphPad Prism (GraphPad Software Inc., San Diego, CA, USA,) software, version 8. 

## 5. Conclusions

In summary, AgNPs, produced using the cyanobacterial species *Synechocystis* sp., were characterized using UV, FTIR spectrophotometry, and TEM, and the nanoparticles’ antibacterial activity against MRSA was demonstrated. The biosynthesized AgNPs have revealed efficiency in the wound healing process in STZ-induced diabetic rat models. The results provided a sufficient support of the wound-healing potency of AgNPs (10 and 30 μg/kg) in excision and incision wound models. Quicker wound closure, increased hydroxyproline, collage and hexamine contents, and a reduced epithelialization period confirmed the wound healing potency of AgNPs. The substantial effect of wound repairing was further supported by angiogenesis factors and enzymatic antioxidant level escalation, as well as the attenuation of inflammatory cytokines.

## Figures and Tables

**Table 1:** Percentage of incision wound contraction, tensile strength (g/s), and hydroxyproline levels (µg/mg) in the different experimental groups.

	Incision Wound Contraction Percentage			Tensile Strength (g/s)	Hydroxyproline Levels (µg/mg)
	7th Day	14th Day	21st Day		
Diabetic negative	37.3 ± 3.67	50.96 ± 3.91	88.49 ± 12.27	0.28 ± 0.1	0.22 ± 0.07
Diabetic positive	50.96 ± 5.93 *	74.7 ± 5.4 *	100 *	0.68 ± 0.71 *	0.47 ± 0.15 *
Diabetic + AgNPs (10 μg/kg)	52.5 ± 8.6 *	85.69 ± 4.5 *^,^#	100 *	0.74 ± 0.32 *^,^#	0.50 ± 0.05 *^,^#
Diabetic + AgNPs (30 μg/kg)	55.7 ± 6.4 *	89.4 ± 6.32 *^,^#	100 *	0.82 ± 0.20 *^,^#	0.59 ± 0.27 *^,^#

All values were expressed as mean ± SD (*n* = 6). * indicates statistically significant from diabetic negative control group, # indicates statistically significant from diabetic positive control group (*p* < 0.05) using one-way ANOVA followed by Tukey’s test as a post hoc analysis.

## References

[1] Diegelmann R.F., Evans M.C. (2004). Wound healing: An overview of acute, fibrotic and delayed healing. Front. Biosci. J. Virtual Libr..

[2] Schäfer M., Werner S. (2008). Oxidative stress in normal and impaired wound repair. Pharmacol. Res..

[3] Rosenberg C.S. (1990). Wound healing in the patient with diabetes mellitus. Nurs. Clin. N. Am..

[4] Ren J., Yang M., Xu F., Chen J., Ma S. (2019). Acceleration of wound healing activity with syringic acid in streptozotocin induced diabetic rats. Life Sci..

[5] Kant V., Gopal A., Kumar D., Pathak N.N., Ram M., Jangir B.L., Tandan S.K., Kumar D. (2015). Curcumin-induced angiogenesis hastens wound healing in diabetic rats. J. Surg. Res..

[6] Rekha P.D., Rao S.S., Sahana T.G., Prabhu A. (2018). Diabetic wound management. Br. J. Community Nurs..

[7] Okonkwo U.A., Chen L., Ma D., Haywood V.A., Barakat M., Urao N., DiPietro L.A. (2020). Compromised angiogenesis and vascular Integrity in impaired diabetic wound healing. PLoS ONE.

[8] Bauer S.M., Bauer R.J., Velazquez O.C. (2005). Angiogenesis, vasculogenesis, and induction of healing in chronic wounds. Vasc. Endovasc. Surg..

[9] Okonkwo U.A., DiPietro L.A. (2017). Diabetes and Wound Angiogenesis. Int. J. Mol. Sci..

[10] Gallagher K.A., Liu Z.J., Xiao M., Chen H., Goldstein L.J., Buerk D.G., Nedeau A., Thom S.R., Velazquez O.C. (2007). Diabetic impairments in NO-mediated endothelial progenitor cell mobilization and homing are reversed by hyperoxia and SDF-1α. J. Clin. Investig..

[11] Weigelt C., Rose B., Poschen U., Ziegler D., Friese G., Kempf K., Koenig W., Martin S., Herder C. (2009). Immune mediators in patients with acute diabetic foot syndrome. Diabetes Care.

[12] Dadpay M., Sharifian Z., Bayat M., Bayat M., Dabbagh A. (2012). Effects of pulsed infra-red low level-laser irradiation on open skin wound healing of healthy and streptozotocin-induced diabetic rats by biomechanical evaluation. J. Photochem. Photobiol. B Biol..

[13] Giacco F., Brownlee M. (2010). Oxidative stress and diabetic complications. Circ. Res..

[14] Choudhury H., Pandey M., Lim Y.Q., Low C.Y., Lee C.T., Marilyn T.C.L., Loh H.S., Lim Y.P., Lee C.F., Bhattamishra S.K. (2020). Silver nanoparticles: Advanced and promising technology in diabetic wound therapy. Mater. Sci. Eng. C Mater. Biol. Appl..

[15] Qayoom A., Aneesha V.A., Anagha S., Dar J.A., Kumar P., Kumar D. (2019). Lecithin-based deferoxamine nanoparticles accelerated cutaneous wound healing in diabetic rats. Eur. J. Pharmacol..

[16] Chinnasamy G., Chandrasekharan S., Koh T.W., Bhatnagar S. (2021). Synthesis, Characterization, Antibacterial and Wound Healing Efficacy of Silver Nanoparticles from *Azadirachta indica*. Front. Microbiol..

[17] Zhang Z., Xin G., Zhou G., Li Q., Veeraraghavan V.P., Krishna Mohan S., Wang D., Liu F. (2019). Green synthesis of silver nanoparticles from Alpinia officinarum mitigates cisplatin-induced nephrotoxicity via down-regulating apoptotic pathway in rats. Artif. Cells Nanomed. Biotechnol..

[18] Ahmed S., Ahmad M., Swami B.L., Ikram S. (2016). A review on plants extract mediated synthesis of silver nanoparticles for antimicrobial applications: A green expertise. J. Adv. Res..

[19] Deljou A., Goudarzi S. (2016). Green Extracellular Synthesis of the Silver Nanoparticles Using Thermophilic Bacillus Sp. AZ1 and its Antimicrobial Activity Against Several Human Pathogenetic Bacteria. Iran. J. Biotechnol..

[20] Mathur P., Jha S., Ramteke S., Jain N.K. (2018). Pharmaceutical aspects of silver nanoparticles. Artif. Cells Nanomed. Biotechnol..

[21] Chung I.M., Park I., Seung-Hyun K., Thiruvengadam M., Rajakumar G. (2016). Plant-Mediated Synthesis of Silver Nanoparticles: Their Characteristic Properties and Therapeutic Applications. Nanoscale Res. Lett..

[22] Naraginti S., Kumari P.L., Das R.K., Sivakumar A., Patil S.H., Andhalkar V.V. (2016). Amelioration of excision wounds by topical application of green synthesized, formulated silver and gold nanoparticles in albino Wistar rats. Mater. Sci. Eng. C Mater. Biol. Appl..

[23] Dkhil M.A., Abdel-Gaber R., Alojayri G., Al-Shaebi E.M., Qasem M.A.A., Murshed M., Mares M.M., El-Matbouli M., Al-Quraishy S. (2020). Biosynthesized silver nanoparticles protect against hepatic injury induced by murine blood-stage malaria infection. Environ. Sci. Pollut. Res. Int..

[24] Pope M.A., Hodge J.A., Nixon P.J. (2020). An Improved Natural Transformation Protocol for the Cyanobacterium *Synechocystis* sp. PCC 6803. Front. Plant. Sci..

[25] Devanesan S., AlSalhi M.S., Balaji R.V., Ranjitsingh A.J.A., Ahamed A., Alfuraydi A.A., AlQahtani F.Y., Aleanizy F.S., Othman A.H. (2018). Antimicrobial and Cytotoxicity Effects of Synthesized Silver Nanoparticles from *Punica granatum* Peel Extract. Nanoscale Res. Lett..

[26] Fathy W., Elyamany K., Tawfik E., Essawy E., Zaki A., Abdel-Hameed M., Hammouda O. (2020). Biosynthesis of silver nanoparticles from *Synechocystis* sp. for using as a flocculent agent with different microalgae strains. Curr. Nanomater..

[27] Hamida R.S., Abdelmeguid N.E., Ali M.A., Bin-Meferij M.M., Khalil M.I. (2020). Synthesis of Silver Nanoparticles Using a Novel Cyanobacteria *Desertifilum* sp. extract: Their Antibacterial and Cytotoxicity Effects. Int. J. Nanomed..

[28] Dietsch M., Behle A., Westhoff P., Axmann I.M. (2021). Metabolic engineering of *Synechocystis* sp. PCC 6803 for the photoproduction of the sesquiterpene valencene. Metab. Eng. Commun..

[29] Aguilera A., Steelheart C., Alegre M., Berdun F., Salerno G., Bartoli C., Pagnussat G., Martin M.V. (2020). Measurement of Ascorbic Acid and Glutathione Content in *Cyanobacterium Synechocystis* sp. PCC 6803. Bio-Protocol.

[30] Rajeshkumar S., Malarkodi C., Paulkumar K., Vanaja M., Gnanajobitha G., Annadurai G. (2014). Algae Mediated Green Fabrication of Silver Nanoparticles and Examination of Its Antifungal Activity against Clinical Pathogens. Int. J. Met..

[31] Aktara M.N., Nayim S., Sahoo N.K., Hossain M. (2019). The synthesis of thiol-stabilized silver nanoparticles and their application towards the nanomolar-level colorimetric recognition of glutathione. New J. Chem..

[32] Hong T., Yin J.-Y., Nie S.-P., Xie M.-Y. (2021). Applications of infrared spectroscopy in polysaccharide structural analysis: Progress, challenge and perspective. Food Chem. X.

[33] Forfang K., Zimmermann B., Kosa G., Kohler A., Shapaval V. (2017). FTIR Spectroscopy for Evaluation and Monitoring of Lipid Extraction Efficiency for Oleaginous Fungi. PLoS ONE.

[34] Huang H., Yang X. (2004). Synthesis of polysaccharide-stabilized gold and silver nanoparticles: A green method. Carbohydr. Res..

[35] Krishnakumar S., Ramanathan G., Hemalatha R. (2017). Polysaccharide Induced Production of Silver Nanoparticles (AG-NPS) and Their Antibacterial Efficacy against Selected Bacterial Pathogens. Int. J. Pharm. Bio. Sci..

[36] Rao C., Trivedi D.d. (2006). Biphasic synthesis of fatty acids stabilized silver nanoparticles: Role of experimental conditions on particle size. Mater. Chem. Phys..

[37] Bakshi K., Liyanage M.R., Volkin D.B., Middaugh C.R. (2014). Fourier transform infrared spectroscopy of peptides. Therapeutic Peptides.

[38] Haris P.I., Chapman D. (1994). Analysis of polypeptide and protein structures using Fourier transform infrared spectroscopy. Methods Mol. Biol..

[39] Reithofer M.R., Lakshmanan A., Ping A.T., Chin J.M., Hauser C.A. (2014). In situ synthesis of size-controlled, stable silver nanoparticles within ultrashort peptide hydrogels and their anti-bacterial properties. Biomaterials.

[40] Ballottin D., Fulaz S., Souza M.L., Corio P., Rodrigues A.G., Souza A.O., Gaspari P.M., Gomes A.F., Gozzo F., Tasic L. (2016). Elucidating Protein Involvement in the Stabilization of the Biogenic Silver Nanoparticles. Nanoscale Res. Lett..

[41] Manam V.K., Subbiah M. (2020). Biosynthesis and Characterization of Silver Nanoparticles from Marine Macroscopic Brown Seaweed *Colpomenia sinuosa* (Mertens ex Roth) Derbes and Solier. J. Adv. Chem. Sci..

[42] Singla R., Soni S., Patial V., Kulurkar P.M., Kumari A., Mahesh S., Padwad Y.S., Yadav S.K. (2017). Cytocompatible Anti-microbial Dressings of Syzygium cumini Cellulose Nanocrystals Decorated with Silver Nanoparticles Accelerate Acute and Diabetic Wound Healing. Sci. Rep..

[43] Vijayakumar V., Samal S.K., Mohanty S., Nayak S.K. (2019). Recent advancements in biopolymer and metal nanoparticle-based materials in diabetic wound healing management. Int. J. Biol. Macromol..

[44] Shrivastava S., Bera T., Roy A., Singh G., Ramachandrarao P., Dash D. (2007). Characterization of enhanced antibacterial effects of novel silver nanoparticles. Nanotechnology.

[45] Dorsett-Martin W.A. (2004). Rat models of skin wound healing: A review. Wound Repair Regen..

[46] Grada A., Mervis J., Falanga V. (2018). Research Techniques Made Simple: Animal Models of Wound Healing. J. Investig. Dermatol..

[47] Rossing P. (2006). Diabetic Nephropathy: Worldwide epidemic and effects of current treatment on natural history. Curr. Diabetes Rep..

[48] Wong K.K.Y., Liu X. (2010). Silver nanoparticles—The real “silver bullet” in clinical medicine?. MedChemComm.

[49] Armstead A.L., Li B. (2011). Nanomedicine as an emerging approach against intracellular pathogens. Int. J. Nanomed..

[50] Franková J., Pivodová V., Vágnerová H., Juráňová J., Ulrichová J. (2016). Effects of silver nanoparticles on primary cell cultures of fibroblasts and keratinocytes in a wound-healing model. J. Appl. Biomater. Funct. Mater..

[51] Younis N.S., El Semary N.A., Mohamed M.E. (2021). Silver nanoparticles green synthesis via cyanobacterium Phormidium sp.: Characterization, wound healing, antioxidant, antibacterial, and anti-inflammatory activities. Eur. Rev. Med. Pharmacol. Sci..

[52] Galkowska H., Wojewodzka U., Olszewski W.L. (2006). Chemokines, cytokines, and growth factors in keratinocytes and dermal endothelial cells in the margin of chronic diabetic foot ulcers. Wound Repair Regen..

[53] Guo S., Dipietro L.A. (2010). Factors affecting wound healing. J. Dent. Res..

[54] Singer A.J., Clark R.A. (1999). Cutaneous wound healing. N. Engl. J. Med..

[55] Siegel R.C. (1976). Collagen cross-linking. Synthesis of collagen cross-links in vitro with highly purified lysyl oxidase. J. Biol. Chem..

[56] Ghaisas M.M., Kshirsagar S.B., Sahane R.S. (2014). Evaluation of wound healing activity of ferulic acid in diabetic rats. Int. Wound J..

[57] Nithya M., Suguna L., Rose C. (2003). The effect of nerve growth factor on the early responses during the process of wound healing. Biochim. Biophys. Acta.

[58] Liu L., Marti G.P., Wei X., Zhang X., Zhang H., Liu Y.V., Nastai M., Semenza G.L., Harmon J.W. (2008). Age-dependent impairment of HIF-1alpha expression in diabetic mice: Correction with electroporation-facilitated gene therapy increases wound healing, angiogenesis, and circulating angiogenic cells. J. Cell. Physiol..

[59] Ferrara N. (2001). Role of vascular endothelial growth factor in regulation of physiological angiogenesis. Am. J. Physiol. Cell Physiol..

[60] Galiano R.D., Tepper O.M., Pelo C.R., Bhatt K.A., Callaghan M., Bastidas N., Bunting S., Steinmetz H.G., Gurtner G.C. (2004). Topical Vascular Endothelial Growth Factor Accelerates Diabetic Wound Healing through Increased Angiogenesis and by Mobilizing and Recruiting Bone Marrow-Derived Cells. Am. J. Pathol..

[61] Lauer G., Sollberg S., Cole M., Krieg T., Eming S.A., Flamme I., Stürzebecher J., Mann K. (2000). Expression and Proteolysis of Vascular Endothelial Growth Factor is Increased in Chronic Wounds. J. Investig. Dermatol..

[62] Falanga V. (2005). Wound healing and its impairment in the diabetic foot. Lancet.

[63] Bao P., Kodra A., Tomic-Canic M., Golinko M.S., Ehrlich H.P., Brem H. (2009). The role of vascular endothelial growth factor in wound healing. J. Surg. Res..

[64] Lambeth J.D. (2004). NOX enzymes and the biology of reactive oxygen. Nat. Reviews. Immunol..

[65] Steiling H., Munz B., Werner S., Brauchle M. (1999). Different types of ROS-scavenging enzymes are expressed during cutaneous wound repair. Exp. Cell Res..

[66] auf dem Keller U., Kümin A., Braun S., Werner S. (2006). Reactive oxygen species and their detoxification in healing skin wounds. J. Investig. Dermatol. Symp. Proc..

[67] Dos Santos J.M., Tewari S., Mendes R.H. (2019). The Role of Oxidative Stress in the Development of Diabetes Mellitus and Its Complications. J. Diabetes Res..

[68] Lan C.C., Liu I.H., Fang A.H., Wen C.H., Wu C.S. (2008). Hyperglycaemic conditions decrease cultured keratinocyte mobility: Implications for impaired wound healing in patients with diabetes. Br. J. Dermatol..

[69] Aliyev E., Sakallıoǧlu U., Eren Z., Açıkgöz G. (2004). The effect of polylactide membranes on the levels of reactive oxygen species in periodontal flaps during wound healing. Biomaterials.

[70] Paglia D.E., Valentine W.N. (1967). Studies on the quantitative and qualitative characterization of erythrocyte glutathione peroxidase. J. Lab. Clin. Med..

[71] Barnes P.J., Karin M. (1997). Nuclear factor-kappaB: A pivotal transcription factor in chronic inflammatory diseases. N. Engl. J. Med..

[72] Long M., Rojo de la Vega M., Wen Q., Bharara M., Jiang T., Zhang R., Zhou S., Wong P.K., Wondrak G.T., Zheng H. (2016). An Essential Role of NRF2 in Diabetic Wound Healing. Diabetes.

[73] Yamasaki K., Edington H.D., McClosky C., Tzeng E., Lizonova A., Kovesdi I., Steed D.L., Billiar T.R. (1998). Reversal of impaired wound repair in iNOS-deficient mice by topical adenoviral-mediated iNOS gene transfer. J. Clin. Investig..

[74] Donnini S., Ziche M. (2002). Constitutive and inducible nitric oxide synthase: Role in angiogenesis. Antioxid. Redox Signal..

[75] Isenberg J.S. (2004). Nitric oxide modulation of early angiogenesis. Microsurgery.

[76] Eming S.A., Wynn T.A., Martin P. (2017). Inflammation and metabolism in tissue repair and regeneration. Science.

[77] Goldberg M.T., Han Y.-P., Yan C., Shaw M.C., Garner W.L. (2007). TNF-α Suppresses α-Smooth Muscle Actin Expression in Human Dermal Fibroblasts: An Implication for Abnormal Wound Healing. J. Investig. Dermatol..

[78] Martinez F.O., Helming L., Gordon S. (2009). Alternative activation of macrophages: An immunologic functional perspective. Annu. Rev. Immunol..

[79] Al-Hasan R.H., Khanafer M., Eliyas M., Radwan S.S. (2001). Hydrocarbon accumulation by picocyanobacteria from the Arabian Gulf. J. Appl. Microbiol..

[80] Bauer A.W., Kirby W.M., Sherris J.C., Turck M. (1966). Antibiotic susceptibility testing by a standardized single disk method. Am. J. Clin. Pathol..

[81] Prasad S.K., Kumar R., Patel D.K., Hemalatha S. (2010). Wound healing activity of Withania coagulans in streptozotocin-induced diabetic rats. Pharm. Biol..

[82] Parmar K.M., Shende P.R., Katare N., Dhobi M., Prasad S.K. (2018). Wound healing potential of Solanum xanthocarpum in streptozotocin-induced diabetic rats. J. Pharm. Pharmacol..

[83] Süntar I., Küpeli Akkol E., Keles H., Yesilada E., Sarker S.D., Arroo R., Baykal T. (2012). Efficacy of *Daphne oleoides* subsp. *kurdica* used for wound healing: Identification of active compounds through bioassay guided isolation technique. J. Ethnopharmacol..

[84] Yadav E., Singh D., Yadav P., Verma A. (2017). Attenuation of dermal wounds via downregulating oxidative stress and inflammatory markers by protocatechuic acid rich n-butanol fraction of *Trianthema portulacastrum* Linn. in wistar albino rats. Biomed. Pharmacother..

[85] Hajji S., Khedir S.B., Hamza-Mnif I., Hamdi M., Jedidi I., Kallel R., Boufi S., Nasri M. (2019). Biomedical potential of chitosan-silver nanoparticles with special reference to antioxidant, antibacterial, hemolytic and in vivo cutaneous wound healing effects. Biochim. Biophys. Acta (BBA)-Gen. Subj..

[86] Woessner J.F. (1961). The determination of hydroxyproline in tissue and protein samples containing small proportions of this imino acid. Arch. Biochem. Biophys..

[87] Elson L.A., Morgan W.T. (1933). A colorimetric method for the determination of glucosamine and chondrosamine. Biochem. J..

[88] Green L.C., Wagner D.A., Glogowski J., Skipper P.L., Wishnok J.S., Tannenbaum S.R. (1982). Analysis of nitrate, nitrite, and [15N]nitrate in biological fluids. Anal. Biochem..

